# The Impact of Lipaemia on the Laboratory Results in a Patient With Diabetes

**DOI:** 10.7759/cureus.94402

**Published:** 2025-10-12

**Authors:** Quintin A van Staden, Minette Steyn, Anne-Cecilia van Marle, Jaco Joubert

**Affiliations:** 1 Department of Haematology and Cell Biology, School of Pathology, Faculty of Health Sciences, University of the Free State, Bloemfontein, ZAF; 2 Department of Haematology and Cell Biology, Universitas Academic Laboratories, National Health Laboratory Service, Johannesburg, ZAF; 3 Department of Chemical Pathology, School of Pathology, Faculty of Health Sciences, University of the Free State, Bloemfontein, ZAF

**Keywords:** haemoglobin (hb), hematocrit, lipaemia, pseudopolycythaemia, type 2 diabetes mellitus

## Abstract

Diabetic dyslipidaemia influences many laboratory results, which would not appear erroneous in the context of diabetic end organ damage, and can lead to inappropriate clinician response and treatment. Alternatively, abnormal results can be missed due to interference, again with potential consequences. Markedly raised haemoglobin levels are not commonly in keeping with diabetic complications and can alert presiding clinicians to lab interference and to treat results with caution. The vast majority of full blood count analyses make use of a spectrophotometric hemolysate method, which is influenced by dyslipidaemia. In this context, the less performed isovolumetric intact red cell spectrophotometric measurement demonstrated the true haemoglobin value. This case emphasises the need for correlation between laboratory healthcare workers and clinicians to interrogate artefactual laboratory results.

## Introduction

Diabetic end organ damage, such as renal failure, commonly results in electrolyte disturbances that require management. Likewise, effects of medications such as insulin also contribute to these irregularities through cellular shift [1]. Other than electrolytes, dyslipidaemia is also a common complication of diabetes, increasing atherosclerosis [2]. Lipaemia is the result of an increase in lipoproteins and chylomicrons, which increases turbidity and is often overlooked as a cause for erroneous laboratory results. Depending on the analytical method used, spectral interference and volume displacement effects can variably influence the measurement of analytes such as creatinine, sodium, and potassium, among others [3]. Haemoglobin levels can be decreased due to an underproduction of erythropoietin in renal injury, and dehydration can cause a relative increase in haemoglobin levels. When haemoglobin is strikingly raised, especially when it doesn't align with the patient's clinical picture, this may point to an interference in laboratory results [4].

## Case presentation

**Figure 1:**
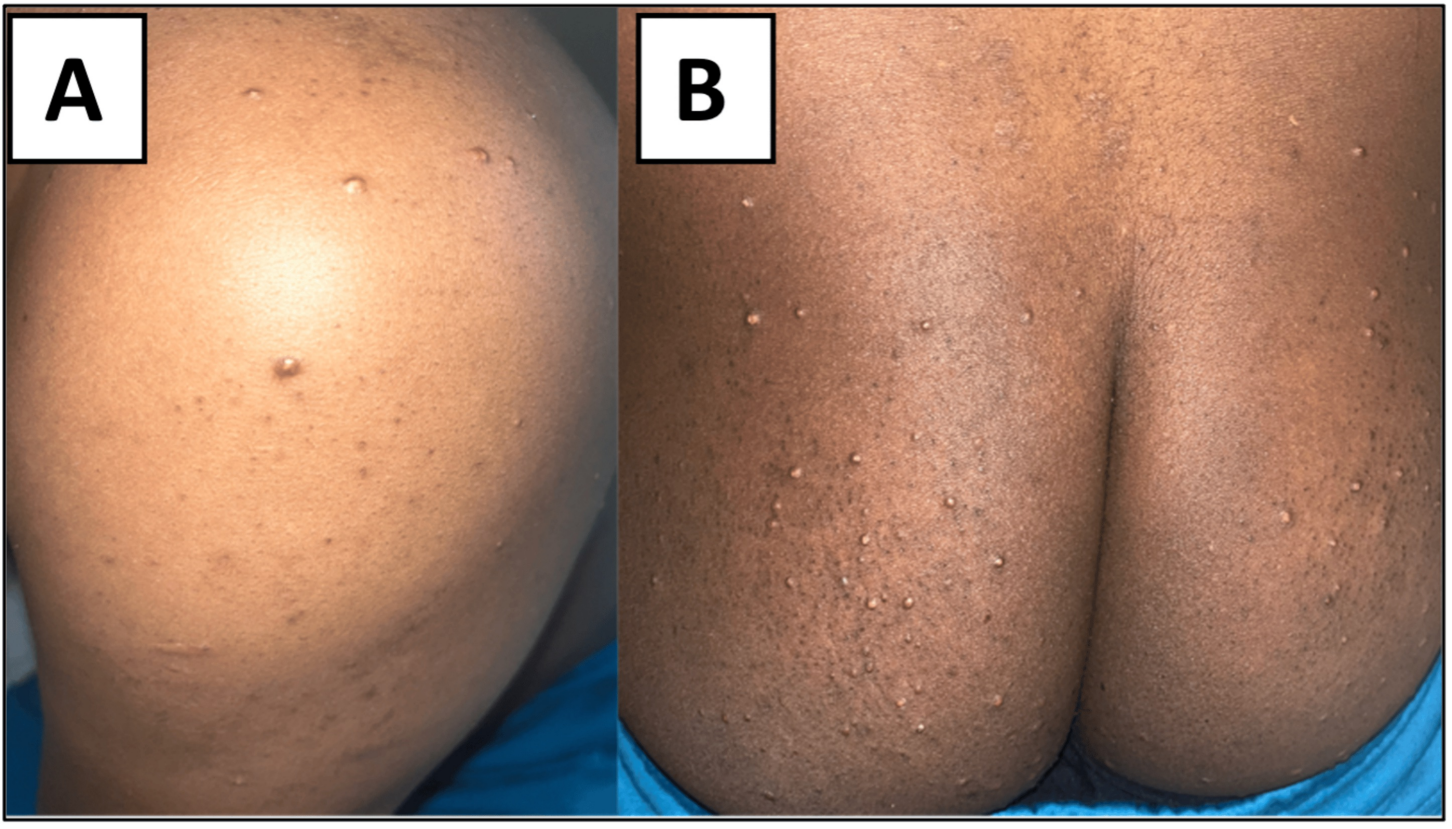
Patient exhibiting widespread eruptive xanthomatosis

A man in his late 30s with a history of hypertension and chronic alcohol use (approximately 40 units per week) presented to a regional hospital with progressive symptoms over one month. He initially developed polyuria and polydipsia, followed by unintentional weight loss of approximately 10 kg over four weeks. In the final week before admission, he experienced increasing upper abdominal pain, nausea, and vomiting, prompting hospital evaluation. He was a non-smoker, had no family history of diabetes and worked as a chef. Clinical examination showed widespread eruptive xanthomatosis, acrochordons, acanthosis nigricans (Figure 1) and generalised abdominal tenderness.

He was transferred to a tertiary hospital and admitted to the endocrinology ward. His initial laboratory work-up (Table 1) revealed a significantly increased haemoglobin (31.3 g/dL) with a normal haematocrit, neutrophilia (11.92 x 10^9^/L), and increased lipase (144 U/L).

**Table 1:** Pertinent laboratory results MCV: Mean Corpuscular Volume.

Parameter	Result	Reference interval
White cell count (x10^9^/L)	13.19	3.92-10.40
Neutrophils (x10^9^/L)	11.92	1.60-6.98
Haemoglobin (x10^12^/L)	31.3	13.4-17.5
MCV (fL)	94.4	83.1-101.6
Haematocrit (L/L)	0.402	0.390-0.510
Platelet count (x10^9^/L)	333	171-388
Sodium (mmol/L)	112	136-145
Potassium (mmol/L)	2.8	3.5-5.1
Creatinine (mmol/L)	<15	64-104
Lipase (U/L)	144	13-60
HbA1c (%)	10.4	>=6.5
Total cholesterol (mmol/L)	40.96	<6.2
Triglyceride (mmol/L)	153.25	<1.7

**Figure 2:**
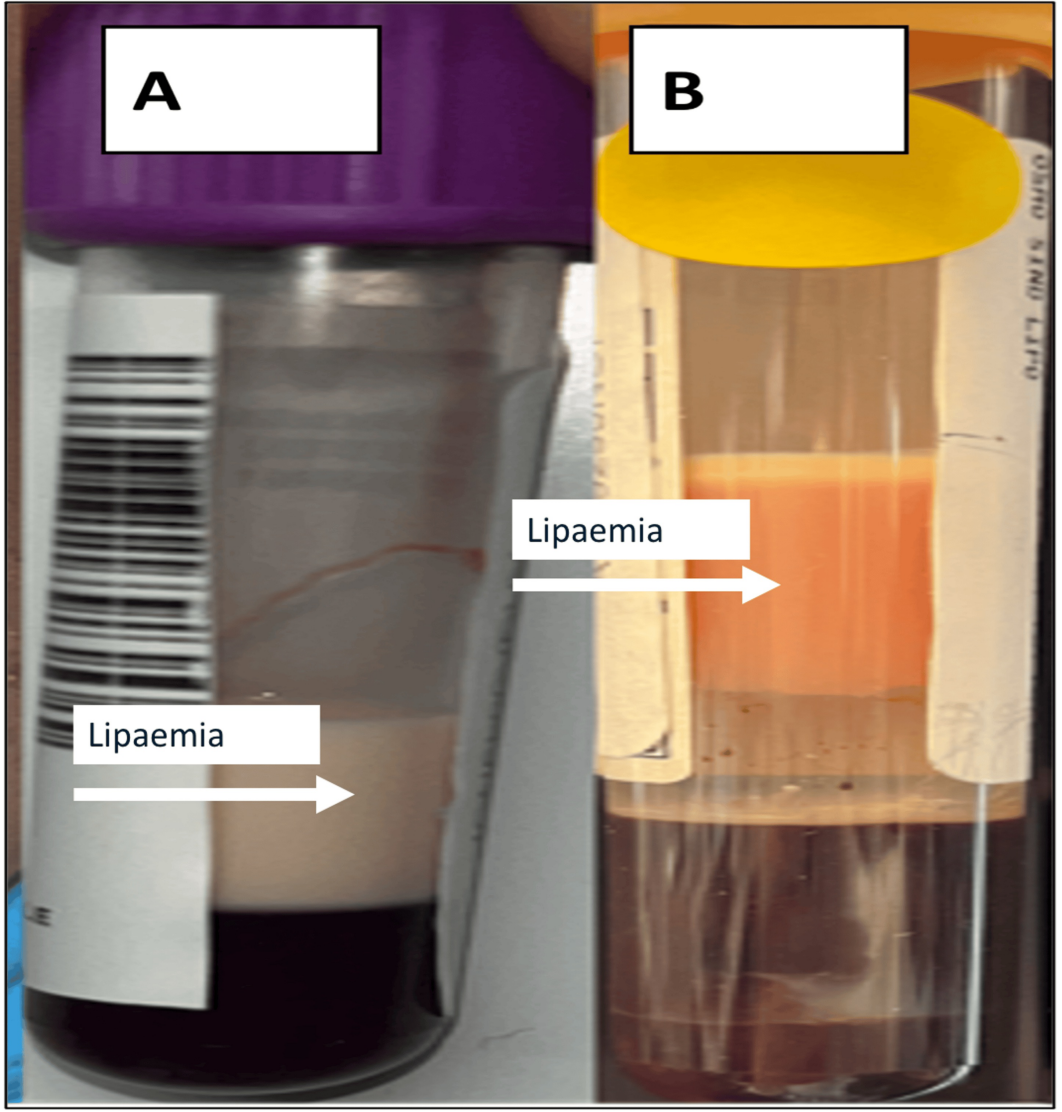
Macroscopic lipaemia

He also exhibited overtly increased total cholesterol (40.96 mmol/L) and triglyceride levels (153.25 mmol/L; Figure 2, macroscopic lipaemia).

**Figure 3:**
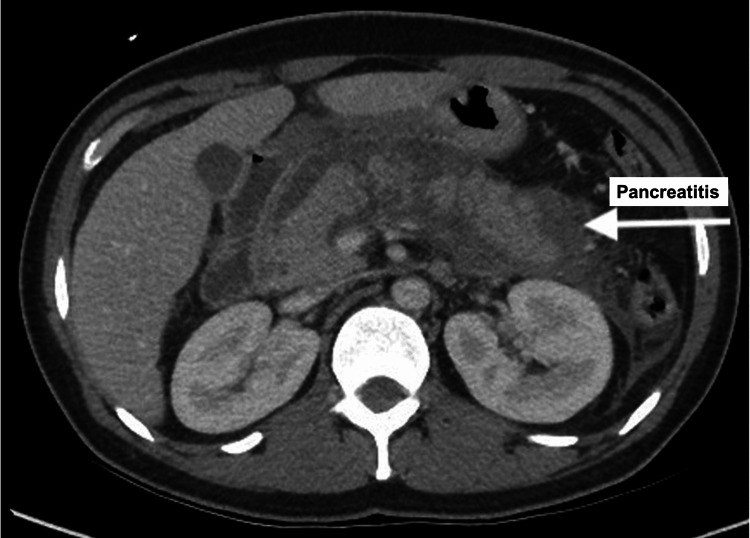
CT scan of pancreatitis without necrosis

The patient had critically low sodium (112 mmol/L) and low potassium levels (2.8 mmol/L). An increased haemoglobin A1C (HbA1c) (10.4%) confirmed diabetes mellitus as the unifying underlying diagnosis secondary to alcohol-induced pancreatitis. A CT scan confirmed pancreatitis without necrosis (Figure 3).

**Figure 4:**
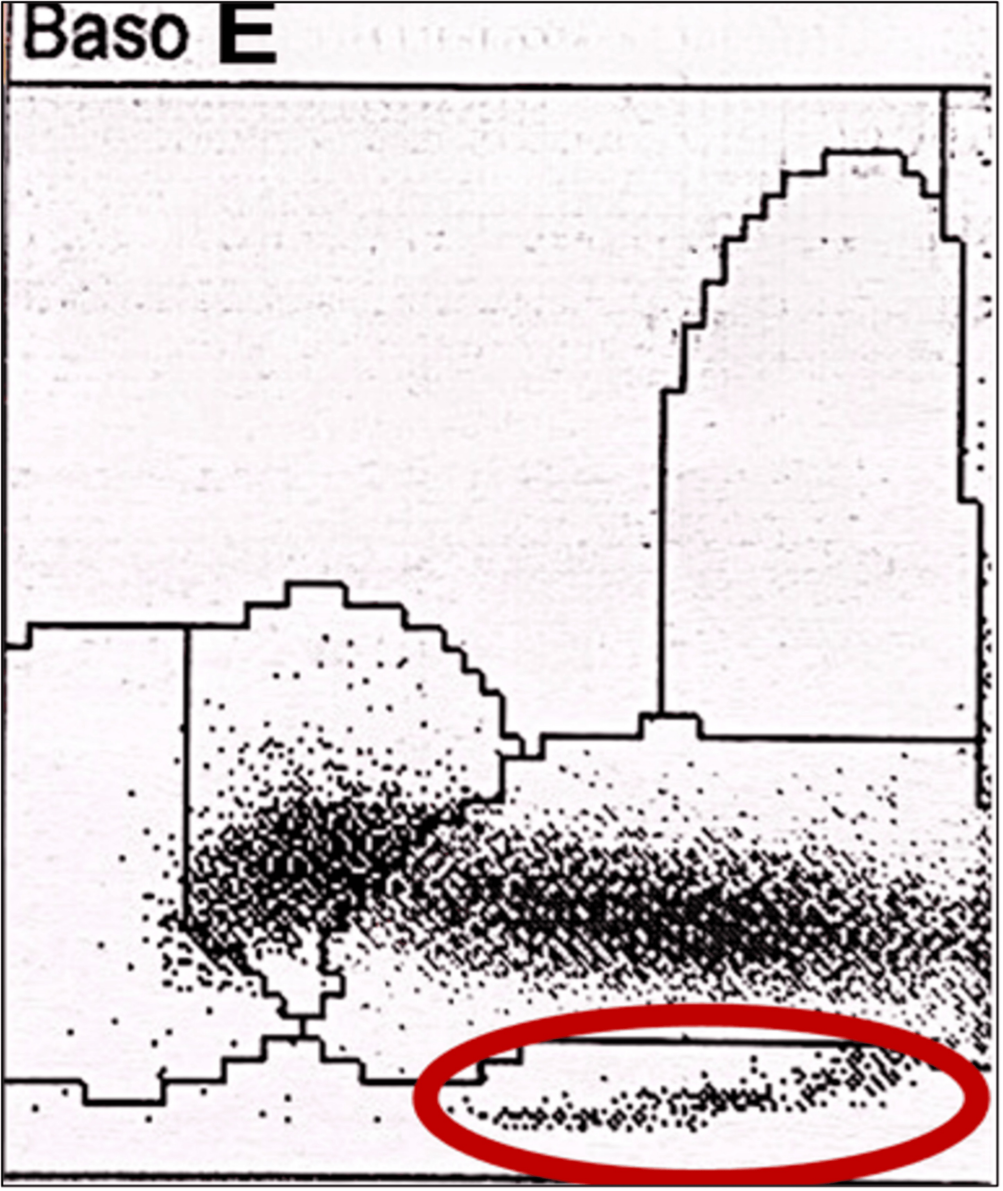
Basophil channel scatter plot, showing the characteristic comma seen with lipaemia

The Advia® 2120i (Siemens Healthineers, Erlangen, Germany) showed a comma shape on the scatter plot (Figure 4). 

The patient was treated with proton pump inhibitors, amlodipine, intravenous fluids, atorvastatin, and fibrates. He was initially placed on a diabetic sliding scale before being initiated on a dual-acting human insulin formulation (Actraphane) with a dose of 44 units in the morning and 24 units at night. His clinical condition and laboratory parameters dramatically improved with adequate glucose control. He was later discharged after diabetic counselling, and asked to follow up at the endocrine clinic. 

## Discussion

The patient’s presentation prompted consideration of several initial differentials, including alcohol-induced pancreatitis, diabetic ketoacidosis, hypertriglyceridaemia-induced pancreatitis, and acute cholecystitis. Alcoholic pancreatitis was plausible given the chronic heavy alcohol use, while diabetic ketoacidosis (DKA) was suggested by the polyuria, polydipsia, and hyperglycaemia. Imaging excluded biliary obstruction, and the combination of clinical features, biochemical results, and radiological findings supported hypertriglyceridaemia-induced pancreatitis secondary to new-onset diabetes mellitus as the final diagnosis.

While the diagnostic tests for diabetes are well known, less considered is the influence of diabetic dyslipidaemia on various laboratory results [5]. There are a number of case studies underlining this principle, with complications from treating dyslipidaemia-related pseudohyponatremia with normal saline being frequently cited and can be fatal [6,7]. In terms of false potassium results due to dyslipidaemia, an ECG can be used to confirm or raise suspicion of result interference [8].

There is also a responsibility on laboratories to identify lipaemic samples and notify clinicians. Management of lipaemic samples is not as well standardised as haemolysed samples, but options include providing warnings to clinicians, cancelling and masking tests, or using methods such as ultracentrifugation to clear lipaemia from samples. However, this method is not readily available [9]. In this case, the markedly increased haemoglobin level performed on the Advia® 2120i guided us and suggested a potential analytical error, with a haemoglobin difference of 17.6 g/dL between the two methods. In the spectrophotometric hemolysate method, red cells are completely lysed and haemoglobin is measured in the resulting solution by absorbance at 540 nm, a process susceptible to interference from lipemia, icterus, or turbidity. In contrast, the isovolumetric intact red-cell spectrophotometric method quantifies haemoglobin directly within undiluted, intact erythrocytes by measuring absorbance per unit cell volume. This avoids dilutional and optical artefacts and provides an in-situ estimate of intracellular haemoglobin concentration [10-12].

Neither dehydration nor common secondary causes of erythrocytosis typically present with such high haemoglobin values and were therefore excluded. This finding was particularly unusual given the normal haematocrit [13,14]. A significant discrepancy was noticed between the direct intracellular haemoglobin measurement (13.7 g/dL), which optically measures red cell complexity in isovolumetrically sphered red cells, and the typical sodium lauryl sulphate spectrophotometric haemolysate method [15]. Spectrophotometry is vulnerable to lipaemic interference due to an increase in turbidity. In marked hyperlipidaemia, lipaemic plasma induces analytical artefacts. Turbidity may cause a spurious elevation of haemoglobin and derived red-cell indices (Mean Corpuscular Haemoglobin (MCH, Mean Corpuscular Haemoglobin Concentration (MCHC)), as well as miscounting of platelets in optical haematology systems. Concurrently, lipaemia exerts a “volume-displacement” and light-scattering effect on diluted chemistry assays, which may produce falsely low readings of chloride, calcium, phosphorus, and magnesium (in addition to sodium), depending on the analyzer and method [16].

In support of the lipaemia-induced pseudopolycythaemia, the basophil channel scatter plot (Figure 4), not routinely published on laboratory reports, showed a characteristic “comma” [17]. The instrument-generated print-out includes this cytogram which is typically only viewed by laboratory staff, including haematologists. Furthermore, measurements using indirect ion selective electrode (ISE) methodology as used on a Cobas 6000® (Roche diagnostics, Germany), and most other automated platforms in clinical laboratory settings, can produce erroneously low sodium and potassium results due to the electrolyte exclusion effect [18]. Since indirect ISE methods measure electrolytes in serum after dilution, they assume a fixed proportion of water in serum. When non-aqueous components such as lipids or proteins are markedly increased, the relative water content decreases, leading to an underestimation of electrolyte concentrations [19]. This may confound hyponatraemia in severe hyperglycaemia or prompt inappropriate potassium replacement during insulin therapy.

## Conclusions

This case emphasises the need to correlate laboratory results with the clinical context of diabetic dysplipidaemia and the importance of engagement between multidisciplinary laboratory specialists and clinicians. Dyslipidaemia is a potential disruptor of laboratory accuracy which introduces the risk of misdirected therapy, particularly when treating increased and decreased sodium and potassium levels. Here, the very high haemoglobin and normal haematocrit provided the critical clue that results were influenced by an underlying interference. This case serves as a reminder to interrogate results and to treat the patient and not only a result.
